# Urinary DNA Extraction in Uro-Oncology and Selected Non-Urological Malignancies: A Narrative Review

**DOI:** 10.3390/jcm15186943

**Published:** 2026-09-08

**Authors:** Bogdan-Petru Tichil, Anamaria Besleaga, Mihaela Laura Vica Matei, Adrian Florea

**Affiliations:** 1 Department of Cell and Molecular Biology, Faculty of Medicine, University of Medicine and Pharmacy “Iuliu Hatieganu”, 400349 Cluj-Napoca, Romania; adrian_a_florea@yahoo.com; 2 Deva Emergency County Hospital, Boulevard 22 Decembrie, Number 58, Deva, Hunedoara County, 330084 Deva, Romania; besleaga.anamaria@elearn.umfcluj.ro; 3 Department of Otorhinolaryngology, University of Medicine and Pharmacy “Iuliu Hatieganu”, 400349 Cluj-Napoca, Romania

**Keywords:** urothelial carcinoma, urinary DNA, real-time PCR, liquid biopsy

## Abstract

Urine is an attractive source of tumor-derived DNA because collection is non-invasive, repeatable, and compatible with large-volume or home-based sampling. Urinary DNA comprises biologically distinct fractions, principally cellular DNA from exfoliated cells and urinary cell-free DNA (cfDNA), which may originate from local release within the urinary tract or from transrenal passage of circulating DNA. Consequently, analytical performance is strongly influenced by urine collection, fractionation, stabilization, extraction, and downstream molecular analysis. This narrative review summarizes evidence from 58 studies addressing urinary DNA extraction and its application in oncology. Analytical studies, including experiments using healthy volunteers, synthetic DNA, and controlled laboratory conditions, demonstrate substantial differences between extraction platforms, particularly in the recovery of short and ultrashort cfDNA fragments. These findings establish analytical performance but should not be interpreted as evidence of clinical superiority. Clinical studies have subsequently demonstrated the feasibility of urinary DNA for mutation detection, methylation analysis, copy-number profiling, surveillance, and treatment-response monitoring, with the strongest evidence currently available in urothelial carcinoma. Applications have also been reported in selected non-urological malignancies. However, methodological standardization, prospective clinical validation, inter-laboratory reproducibility, control of biological and clinical confounders, demonstration of clinical utility, and cost-effectiveness remain necessary before urinary DNA analysis can be incorporated routinely into oncological practice.

## 1. Introduction

Liquid biopsy provides access to tumor-derived nucleic acids without repeated tissue sampling. Although plasma cfDNA remains the most extensively studied analyte, urine offers important practical advantages: collection is completely non-invasive, repeatable, does not require trained personnel, and allows relatively large specimen volumes.

Early studies demonstrated that human genomic DNA recovered from urine could support PCR and genotyping despite variability related to storage, DNA quality, and target length [1,2,3]. Methodological advances subsequently introduced magnetic-particle capture, separate isolation of cellular and cell-free fractions, nanoparticle-based extraction, and protocols applicable to long-term stored specimens [4,5,6,7]. Conventional phenol–chloroform extraction, commercial kits, Chelex-based protocols, and automated extraction systems further demonstrated that urinary DNA can support PCR, genotyping, sequencing, and biobanking applications [8,9,10,11,12].

These studies established a fundamental principle for urinary molecular diagnostics: analytical performance depends not only on the biomarker and detection platform but also on how DNA is collected, preserved, fractionated, and extracted.

These biological distinctions are central to extraction-method selection because a method optimized for intact genomic DNA may perform poorly for ultrashort transrenal DNA, while a method designed for very short cfDNA may not be optimal for cellular DNA-based methylation or sequencing assays.

Definitions and urinary DNA fractions: For consistency, several terms require explicit definition. In this review, urinary cellular DNA or urine pellet DNA refers predominantly to genomic DNA recovered from cells collected by centrifugation of urine. Urinary supernatant DNA refers to DNA recovered from the cell-depleted supernatant; depending on centrifugation efficiency, this fraction may contain both true extracellular DNA and residual cellular DNA. Urinary cell-free DNA (cfDNA) refers specifically to extracellular DNA present in the cell-depleted urine fraction. Urinary tumor DNA denotes the tumor-derived component of urinary DNA irrespective of whether it originates from local tumor shedding or the circulation. Transrenal DNA refers to circulating DNA that reaches urine after passage through the renal filtration barrier and is frequently highly fragmented. The term circulating tumor DNA (ctDNA) is reserved for the tumor-derived component of circulating cfDNA; when the origin of tumor DNA detected in urine cannot be demonstrated to be transrenal, the broader term urinary tumor DNA is preferable.

## 2. Methods

A targeted narrative literature search was performed in PubMed/MEDLINE, Scopus, Web of Science Core Collection, and Google Scholar from database inception to 17 August 2026. Search terms were used individually and in combination and included “urine”, “urinary”, “DNA extraction”, “DNA isolation”, “cell-free DNA”, “cfDNA”, “circulating tumor DNA”, “ctDNA”, “transrenal DNA”, “urinary tumor DNA”, “DNA methylation”, “cancer”, “oncology”, “urothelial”, “bladder”, “upper tract urothelial”, “prostate”, “renal”, “colorectal”, “hepatocellular”, “endometrial”, “lung”, and “oropharyngeal”. Reference lists of relevant articles were also screened to identify additional eligible studies.

Studies were considered eligible when they investigated human urine and either: evaluated DNA extraction, purification, fractionation, preservation, storage, or pre-analytical processing; or applied extracted urinary DNA to oncological diagnosis, surveillance, molecular profiling, or treatment monitoring. Selected non-oncological human studies were retained when they provided directly relevant methodological information regarding urinary DNA extraction, DNA recovery, genotyping, or downstream assay compatibility. Studies primarily addressing microbial or parasitic DNA, microbiome analysis, forensic identification, animal samples, RNA-only biomarkers, or non-urinary specimens were excluded. Only articles available in English with sufficient methodological information were considered. Because the objective was a narrative rather than a systematic review, no formal meta-analysis or risk-of-bias assessment was performed. The final literature set comprised 59 studies. For interpretation, the evidence was divided into two broad categories: analytical/laboratory studies assessing DNA recovery and pre-analytical performance, and clinical/translational studies evaluating diagnostic, surveillance, prognostic, or treatment-monitoring applications. This distinction was maintained throughout the review because superior analytical DNA recovery does not necessarily translate into superior clinical performance.

## 3. Results

### 3.1. Urinary DNA Fractions and Extraction Requirements

Urinary DNA is not a homogeneous analyte. Cellular genomic DNA originates from exfoliated urothelial, renal tubular, squamous, inflammatory, and malignant cells, whereas urinary cfDNA derives from local cellular degradation or from circulating DNA that crosses the renal filtration barrier.

Cellular DNA is generally longer and can be concentrated by centrifugation before conventional genomic DNA extraction. In contrast, cfDNA is less abundant, more fragmented, and highly susceptible to nuclease degradation. Delayed processing may additionally cause cellular lysis, increasing background genomic DNA and reducing the relative abundance of tumor-derived cfDNA.

Commercial extraction studies demonstrate considerable kit-dependent and inter-individual variation in urinary cfDNA recovery [13]. Obtaining sufficient genomic DNA for demanding analyses such as genome-wide methylation profiling can also be difficult even after increasing urine volume or changing extraction systems [14]. Pre-analytical conditions are particularly important for methylation assays because preservation and storage affect amplifiable DNA, while subsequent bisulfite treatment causes additional DNA degradation [15].

Thus, the optimal extraction strategy depends on whether the intended analyte is cellular DNA, conventional cfDNA, or highly fragmented transrenal DNA.

### 3.2. Analytical and Laboratory Evidence: Extraction Chemistry and Fragment-Size Selectivity

The studies discussed in this section primarily address analytical recovery rather than clinical diagnostic performance. Several used healthy volunteers, synthetic DNA fragments, spiked urine, or small experimental cohorts; therefore, differences in yield or fragment recovery should not be interpreted as evidence that one extraction method provides superior diagnostic accuracy in patients. This is supported especially well by Oreskovic et al., whose study explicitly compared recovery of 25–150-nt fragments under analytical conditions rather than comparing cancer diagnostic outcomes [16].

Fragment-size bias is one of the most important determinants of urinary cfDNA extraction efficiency. Oreskovic et al. compared several commercial and experimental methods using fragments of 25–150 nucleotides and demonstrated marked differences in recovery. Hybridization capture and Q-Sepharose achieved substantially greater recovery than several conventional approaches, whereas QIAamp and MagMAX showed limited efficiency for very short fragments [16].

Preservation is closely linked to extraction performance. Li et al. demonstrated that delayed storage can alter urinary cfDNA integrity and allele fractions, whereas dedicated collection tubes better preserve the original cfDNA profile [17]. Lee et al. compared four commercial urinary cfDNA extraction kits and showed that each had distinct advantages: QIAamp and MagMAX efficiently recovered broader fragment ranges, Norgen preferentially recovered 50–100-bp fragments with relatively low genomic-DNA contamination, and Quick-DNA Urine permitted greater input volumes at lower cost [18].

Sampling conditions further influence recovery. Augustus et al. showed that urine volume, timing of collection, preservatives, storage, and centrifugation significantly affect cfDNA yield and genomic-DNA contamination [19]. Lin et al. similarly demonstrated kit-dependent fragment profiles and showed that additional cleanup could improve PCR amplification efficiency, emphasizing that total extraction yield is not equivalent to amplifiable DNA yield [20].

Sequence-specific hybridization capture represents a more selective approach. Oreskovic and Lutz reported near-complete recovery of short target fragments under experimental conditions and detection at approximately 0.5 copies/mL [21]. Such approaches transform extraction from passive purification into selective molecular enrichment.

### 3.3. Analytical and Laboratory Evidence: Pre-Analytical Stabilization and Urine Processing

Pre-analytical handling should be considered an integral component of urinary DNA extraction. Urine has variable pH, salt concentration, cellular content, and high nuclease activity; therefore, delays in stabilization may simultaneously degrade cfDNA and increase contaminating genomic DNA through cellular lysis.

Dedicated preservatives have improved sample stability. Jordaens et al. demonstrated preservation of cellular integrity and cfDNA under transport-relevant conditions, whereas Nel et al. showed that unpreserved cfDNA could become essentially unrecoverable, while stabilization maintained successful ddPCR detection [22,23].

Urine volume is particularly important for low-abundance tumor DNA. Ruppert et al. demonstrated improved target recovery with increasing specimen volume and suggested that processing more than small urine volumes becomes important when mutant allele frequencies are below 1% [24].

Fragment size is equally critical. Bhambhani et al. demonstrated that transrenal tumor DNA from non-urological malignancies is frequently ultrashort, with substantial enrichment below 50 bp. Analysis restricted to these fragments enhanced tumor-associated copy-number signals, implying that plasma-oriented extraction methods designed around approximately 160-bp cfDNA may discard clinically relevant urinary DNA [25].

More recent studies have emphasized normalization and complete workflow standardization. Sandberg et al. demonstrated method-dependent cfDNA recovery using spike-in controls [26], while Eberhard et al. showed rapid degradation in unstabilized urine but preservation of DNA compatible with digital PCR and next-generation sequencing after appropriate stabilization [27]. Mauger et al. extended these findings to methylation and fragmentomic analyses, showing that inadequate stabilization may alter not only DNA quantity but also biologically informative molecular patterns [28].

Collectively, these studies demonstrate that stabilization, centrifugation, storage, and extraction form a continuous analytical workflow. However, their evidence should be interpreted according to study design. For example, Lee et al. compared four extraction kits in urine from only 10 healthy individuals, whereas several other pre-analytical studies relied on small experimental cohorts or synthetic targets [18,19,23,26,27,28]. Lee et al. demonstrated clear differences in short-fragment recovery among commercial kits, but these findings establish analytical performance rather than comparative diagnostic superiority in cancer patients [18]. Similarly, Augustus et al. included 39 healthy volunteers and 14 patients with metastatic malignancies but divided these participants among multiple small pre-analytical experiments; the authors themselves emphasized that their results required validation in larger populations [19]. Therefore, extraction-method comparisons should be regarded as analytical evidence that informs the design of clinical assays rather than as direct evidence favoring one method for cancer diagnosis or surveillance.

### 3.4. Biological Basis of Urinary Tumor DNA

Tumor DNA may reach urine by local shedding or transrenal passage. Su et al. characterized a high-molecular-weight, predominantly cellular fraction and a smaller 150–250-bp non-cell-associated fraction. Mutated KRAS matching colorectal tumor tissue was enriched in the low-molecular-weight fraction, supporting a circulating origin [29].

Selective depletion of high-molecular-weight DNA using carboxylated magnetic beads subsequently improved detection of mutated KRAS, demonstrating that reducing background wild-type DNA may be as important as maximizing total DNA recovery [30]. Detection of matching KRAS mutations in urine, plasma, and serum further established urine as a potential source of systemic tumor-derived DNA [31].

These observations provided the biological foundation for modern fragment-selection and transrenal cfDNA strategies.

### 3.5. Clinical Evidence in Urothelial Carcinoma

Urothelial carcinoma is the most developed clinical application of urinary DNA because malignant cells and tumor-derived DNA are released directly into the urinary tract. In contrast to the analytical studies described above, the following investigations evaluated urinary DNA in patients with cancer or clinically relevant control groups. Their outcomes include diagnostic sensitivity and specificity, concordance with tumor tissue, recurrence detection, and treatment-response monitoring. Nevertheless, direct comparison of reported diagnostic performance between studies remains difficult because urine volume, fraction, preservation, extraction chemistry, target genes, analytical platform, disease stage, and control populations differ substantially.

Early studies demonstrated FGFR3 mutations in urine sediment DNA and showed that molecular analysis could complement cytology, particularly in low-grade tumors [32]. Comparison of sediment and supernatant subsequently indicated that cell-free supernatant DNA may provide greater sensitivity in some patients [33]. However, biomarker prevalence must also be considered: Wang et al. reported low FGFR3 mutation frequency among Han Chinese patients, while TERT-based markers were more informative [34].

Multiplex sequencing expanded the number of simultaneously detectable alterations. Ward et al. demonstrated successful next-generation sequencing of TERT, FGFR3, PIK3CA, TP53 and other genes from urine cell-pellet DNA despite limited DNA quantities in some samples [35]. Togneri et al. found higher tumor genomic burden in urinary cfDNA than cellular DNA in their cohort [36], while Lee et al. demonstrated that both urinary cfDNA and exosomal DNA can contain tumor-associated mutations and copy-number alterations [37].

Highly sensitive ddPCR has further increased diagnostic potential. Russo et al. reported strong concordance between tumor and urinary cfDNA TERT promoter status [38], whereas Stasik et al. found higher TERT mutation detection in urinary sediment than cfDNA [39]. These differing results emphasize that the optimal urinary fraction depends on tumor biology, extraction method, and analytical platform.

In upper-tract urothelial carcinoma, Hayashi et al. detected TERT C228T/C250T and FGFR3 S249C mutations in urinary cfDNA; combining molecular analysis with cytology achieved 78.6% sensitivity and 96.0% specificity [40]. Broader sequencing approaches demonstrated clinically relevant mutations in both urinary cell-pellet DNA and cfDNA [41], while Ou et al. reported high diagnostic performance from both cellular and cell-free urinary fractions [42].

Methylation analysis provides a complementary strategy. Hentschel et al. compared full-void urine, sediment, and supernatant and found diagnostically informative methylation signals in all fractions, although the pellet performed best in their cohort [43]. Hayashi et al. subsequently reported approximately 69% sensitivity for urinary cfDNA hotspot analysis, increasing to approximately 85% when combined with cytology; higher TERT C228T allele fractions were also associated with recurrence [44].

Urinary tumor DNA does not originate exclusively from direct shedding. Hentschel et al. demonstrated tumor-associated methylation and mutations in urine obtained without direct tumor contact, confirming contributions from both local shedding and transrenal excretion [45].

TERT promoter mutation testing using ddPCR has additionally shown potential for diagnosis and surveillance [46], while urinary-cell DNA methylation assays have demonstrated utility for detection and longitudinal recurrence monitoring [47].

A major emerging application is treatment monitoring. Christensen et al. detected tumor DNA before neoadjuvant chemotherapy in 89% of urine supernatants and 85% of urinary pellets compared with 43% of plasma samples. Changes in urinary tumor DNA during treatment were associated with response and outcome [48].

More recently, long-read sequencing of urinary cell-pellet DNA has enabled direct genome-wide methylation and copy-number profiling [49], while a two-gene urinary methylation assay achieved 85.2% sensitivity and 90.0% specificity for urothelial carcinoma and an AUC of 0.979 for recurrence detection [50].

One bladder-cancer study also evaluated DNA associated with urinary extracellular vesicles and demonstrated that this fraction can contain somatic mutations and copy-number alterations representative of tumor tissue [37]. Because extracellular-vesicle isolation and extracellular-vesicle-associated DNA extraction were not systematically investigated across the studies included in this review, this analyte should currently be regarded as an exploratory subcategory rather than one of the principal urinary DNA extraction frameworks considered here.

Overall, clinical studies indicate that both urinary cellular DNA and cfDNA can provide oncologically relevant information, but current evidence does not establish a universally superior urine fraction. The relative performance of pellet and supernatant DNA varies according to the biomarker, extraction procedure, disease setting, and downstream assay. Accordingly, analytical superiority in DNA recovery should not be equated with clinical superiority, and prospective head-to-head studies using standardized collection and extraction protocols remain necessary.

Hentschel et al., for example, found the pellet most representative for their methylation assay but explicitly acknowledged that the study was sufficiently powered for a technical comparison rather than strong conclusions regarding diagnostic accuracy [43].

### 3.6. Clinical Evidence in Selected Non-Urothelial Malignancies

The value of urinary DNA extends beyond urothelial carcinoma. Crisafulli et al. demonstrated that low-molecular-weight transrenal tumor DNA from metastatic colorectal cancer can support whole-exome sequencing, highlighting the importance of extraction methods that preserve short circulating fragments [51].

In prostate cancer, urinary cfDNA integrity has shown diagnostic potential [52], while whole-genome sequencing has identified copy-number alterations, including androgen-receptor amplification, and treatment-associated genomic changes [53].

Mutation-enrichment sequencing has also enabled detection of very low-level KRAS mutations in urinary cfDNA from patients with advanced cancers, with large urine volumes improving analytical sensitivity [54].

In renal tumors, Smith et al. demonstrated that renal-cell carcinoma is generally characterized by low cfDNA shedding; nevertheless, personalized analysis identified tumor-derived urinary cfDNA in selected patients and supported its potential for longitudinal monitoring [55].

DNA methylation may provide another means of amplifying disease-specific signals. Van den Helder et al. demonstrated endometrial cancer-associated methylation in full-void urine, sediment, and supernatant, with strong correlations between fractions and particularly favorable diagnostic performance in full-void urine [56]. The ability to use unfractionated urine is clinically attractive because it reduces pre-processing requirements, although the optimal strategy may depend on the assay.

Bach et al. investigated urine-based DNA methylation for colorectal cancer. Methylation analysis of urine supernatant identified elevated SEPT9 and other cancer-associated signals, while a SEPT9/SDC2 combination detected up to 70% of colorectal cancers at 86% specificity in their cohort [57].

Urine-derived tumor DNA has also been investigated for hepatocellular carcinoma. Kim et al. evaluated a urinary DNA panel incorporating TP53 mutation and methylated RASSF1A and GSTP1 in a large screening cohort. Although serum alpha-fetoprotein remained more sensitive overall, the urinary DNA panel detected a proportion of cancers with low alpha-fetoprotein and improved early-stage detection when used in combination with serum testing [58].

These studies collectively indicate that extraction strategies for non-urological cancers should prioritize large starting volumes, efficient recovery of very short fragments, prevention of nuclease-mediated degradation, and highly sensitive downstream assays. Evidence outside urothelial carcinoma remains more heterogeneous and generally less mature. Many studies are feasibility or proof-of-concept investigations with small cohorts, and the low abundance of transrenal tumor DNA makes analytical sensitivity particularly dependent on urine volume, stabilization, fragment-size recovery, and downstream assay design. These results therefore demonstrate biological and technical feasibility but should not yet be interpreted as evidence supporting routine clinical implementation.

### 3.7. Practical Selection of Urinary DNA Extraction Methods

The accumulated literature does not support one universally optimal urinary DNA extraction method. Instead, the procedure should be selected according to the biological and analytical target.

For urinary cellular or sediment DNA, conventional genomic-DNA spin columns, magnetic-particle capture, or automated extraction systems are appropriate when adequate numbers of exfoliated cells are present. These approaches are particularly suitable for local urothelial tumors, germline genotyping, long-fragment PCR, methylation assays requiring substantial DNA input, and sequencing applications requiring relatively intact DNA [1,2,3,4,5,6,7,8,9,10,11,12].

For conventional urinary cfDNA, commercial cfDNA-specific kits provide standardized processing but differ substantially in their fragment-size recovery. Methods optimized for broad cfDNA recovery are appropriate for many ddPCR and NGS applications, but the expected target size must be considered [13,14,15,16,17,18,19,20].

For ultrashort transrenal tumor DNA, standard silica extraction may be inadequate. Anion-exchange approaches, carefully optimized magnetic-bead systems, fragment-specific enrichment, or hybridization capture can improve recovery of short targets and reduce dilution by high-molecular-weight wild-type DNA [16,21,25,26]. For methylation analysis, the priority extends beyond initial extraction yield because conversion and library preparation can produce additional DNA loss. Preservation of fragment composition before extraction is therefore particularly important [5,14,15,17,22,23,27,28].

For high-throughput clinical implementation, magnetic extraction and automated systems provide advantages in reproducibility, reduced hands-on time, and scalability [4,6,12,18]. However, automation should not be assumed to compensate for inappropriate fragment-size selectivity.

The most important distinction is between cellular/sediment DNA and urinary cfDNA. Cellular DNA is relatively long and abundant and can usually be extracted effectively using conventional silica columns, magnetic particles, Chelex, or organic methods. These approaches are particularly useful for mutation analysis, methylation assays, and sequencing of exfoliated urothelial cells [1,2,3,4,5,6,7,8,9,10,11,12]. By contrast, urinary cfDNA is dilute and highly fragmented, and transrenal tumor DNA may contain a substantial proportion of fragments below 50–100 bp. Therefore, extraction methods optimized for conventional genomic DNA or plasma cfDNA may lose clinically relevant urinary tumor DNA. Comparative studies have shown marked differences among commercial systems, with some magnetic or specialized urine kits performing better for short fragments [13,16,17,18,19,20,25]. For very short and low-abundance tumor DNA, Q-Sepharose, optimized magnetic-bead approaches, high-molecular-weight DNA depletion, and sequence-specific hybridization capture may provide superior recovery [Table 1]. Hybridization capture is particularly sensitive because it simultaneously extracts and enriches the molecular target [16,21,30]. Finally, extraction should be considered together with urine preservation and preprocessing. Delayed processing, nuclease activity, inappropriate storage, cellular lysis, and centrifugation can alter the DNA population before extraction even begins. Consequently, the optimal clinical workflow is not simply the kit producing the greatest total DNA yield [15,17,19,22,23,24,25,26,27,28].

Cost is an important consideration when selecting a urinary DNA extraction strategy, particularly for large-scale screening or longitudinal surveillance. Simple approaches such as Chelex-based or conventional organic extraction generally have low reagent requirements but involve more manual processing and may provide lower purity or limited compatibility with advanced molecular assays. Commercial silica-column and magnetic-bead systems are more expensive but offer greater standardization and compatibility with PCR, ddPCR, and next-generation sequencing. Lee et al. reported study-specific costs of approximately $2.7 per ng of recovered cfDNA for the Quick-DNA Urine kit, $5.0/ng for MagMAX, and $7.6/ng for the QIAamp Circulating Nucleic Acid kit [18]. These values reflect the kit prices, experimental conditions, urine volumes, DNA yields, and cost assumptions used in that particular study and should not be interpreted as universal or current market costs. More specialized approaches, including sequence-specific hybridization capture and automated extraction platforms, may involve higher reagent or equipment expenditure but can be justified when recovery of very short or low-abundance tumor DNA is required. Future cost-effectiveness analyses should therefore consider not only the cost per extraction but also labor, sample volume, extraction efficiency, clinically relevant fragment recovery, assay failure rates, downstream molecular costs, and ultimately the clinical information obtained per test.

### 3.8. Limitations and Standardization

The principal limitation of the current literature is methodological heterogeneity. Studies differ in urine collection timing, input volume, centrifugation, fractionation, preservation, storage temperature, processing delay, freeze–thaw exposure, extraction chemistry, elution volume, DNA quantification, and downstream assay.

These variables may substantially influence apparent biomarker performance. Higher DNA concentrations after delayed processing, for example, may reflect genomic DNA released from lysed cells rather than preservation of genuine cfDNA. Conversely, apparently low urinary tumor-DNA concentrations may result from poor recovery of short fragments.

Biological and clinical confounders represent an additional source of variability that has received comparatively limited attention. Hematuria may increase the quantity of background DNA derived from blood cells and thereby alter the relative abundance of tumor-derived molecules. In the study by Christensen et al., leukocytes, nitrite, protein, and erythrocytes were associated with urinary cfDNA concentration, while erythrocyte levels were also associated with tumor-DNA positivity and tumor-DNA levels [48]. Similarly, benign hematuria can influence urinary methylation profiles and may complicate comparisons between malignant and non-malignant populations [43]. Urinary tract infection and inflammation may alter leukocyte content, cellular turnover, bacterial burden, and nuclease activity. Hydration status can change urine concentration and consequently measured DNA concentration per milliliter, while renal function may potentially influence the passage and recovery of transrenal DNA. Recent cystoscopy, catheterization, transurethral surgery, biopsy, or other urinary instrumentation may also increase epithelial and blood-cell shedding. These variables should therefore be documented and, where possible, controlled in prospective clinical validation studies. Christensen et al. directly observed associations between dipstick erythrocytes/leukocytes and cfDNA/tumor-DNA measurements. Hentschel et al. similarly noted that background DNA in benign hematuria could influence methylation results.

The need for methodological harmonization has recently been addressed directly by Ward et al. in the article “Unlocking the potential of urine-based liquid biopsy through improved reporting and standardization.” The authors highlight an important paradox in the current urinary liquid-biopsy literature: although tumor-derived urinary DNA has demonstrated substantial potential for profiling urological malignancies and, through transrenal cfDNA, cancers at distant anatomical sites, translation into clinical practice remains limited by inconsistent methodology, incomplete reporting of pre-analytical variables, and insufficient large-scale validation [59]. The article therefore shifts the emphasis from identification of additional urinary biomarkers toward reproducibility of the complete experimental workflow.

To address this problem, Ward et al. proposed the Minimal Urine Methods in Experiments (MUMIE) framework [59]. Rather than prescribing a single extraction kit or processing protocol, MUMIE is intended to ensure that the variables capable of influencing urinary DNA measurements are reported sufficiently to permit replication and comparison between studies. These include characteristics of urine collection and processing, specimen volume, preservation and storage, the urine fraction analyzed, DNA extraction methodology, and other relevant pre-analytical and analytical variables. This approach is particularly appropriate for urinary cfDNA because differences in collection, centrifugation, storage and extraction can alter total DNA concentration, genomic-DNA contamination, fragment-size distribution and the relative abundance of tumor-derived molecules. The MUMIE proposal therefore complements the findings of the analytical studies reviewed here, which demonstrate that apparently small methodological differences can substantially influence measured DNA recovery.

A further development toward formal standardization is ISO 18704:2026, “Molecular in vitro diagnostic examinations—Requirements and recommendations for pre-examination processes for urine and other body fluids—Isolated cell-free DNA” [60]. Published in February 2026, the standard applies to cfDNA obtained from non-blood body fluids including urine, pleural effusions, ascites, cerebrospinal fluid and saliva. It specifies requirements and recommendations covering specimen collection, handling, transport, reception, storage, processing before cfDNA isolation, cfDNA isolation itself, assessment of isolated cfDNA quantity and quality, documentation, and subsequent storage. The standard specifically recognizes that inadequate pre-examination handling can cause both degradation of native cfDNA and release of genomic DNA from nucleated cells, thereby altering the original cfDNA profile [Table 2].

MUMIE and ISO 18704:2026 should therefore be viewed as complementary rather than competing approaches. MUMIE was developed primarily to improve transparency and completeness of reporting in urinary DNA biomarker research, whereas ISO 18704:2026 provides formal requirements and recommendations for the pre-examination phase. Together, they provide a contemporary framework for moving urinary DNA studies from heterogeneous proof-of-concept experiments toward reproducible analytical validation and, ultimately, clinical implementation. Importantly, neither framework removes the need for prospective validation of individual biomarkers or extraction systems; standardized handling makes such comparisons more reliable but does not itself establish diagnostic or clinical superiority.

A further limitation is that many extraction comparisons are based on healthy volunteers, synthetic DNA, spike-in experiments, or small patient cohorts. Such designs are appropriate for measuring extraction efficiency, size bias, PCR inhibition, and stability, but they cannot demonstrate that an extraction technique improves sensitivity, specificity, recurrence detection, survival prediction, or patient outcomes. Analytical validation and clinical validation should therefore be considered separate stages of assay development.

Consistent with the principles emphasized by Ward et al., the MUMIE framework and ISO 18704:2026, future studies should comprehensively document urine collection type and timing, collected and processed volume, stabilization and transport conditions, storage duration and temperature, centrifugation and fractionation procedures, intended DNA fraction, extraction methodology, elution conditions, DNA quantity and quality assessment, fragment-size distribution, downstream assay input and analytical detection threshold [59,60].

Fragment-specific recovery should remain a central validation endpoint, and spike-in controls may improve normalization between samples and laboratories [26]. However, technical standardization alone will not be sufficient. Prospective multicenter validation, inter-laboratory reproducibility, predefined analytical thresholds, clinically appropriate comparator groups, demonstration of incremental clinical utility over established diagnostic pathways, and formal cost-effectiveness analyses are also required before urinary DNA assays can be implemented routinely.

## 4. Conclusions

Urinary DNA represents a promising but technically complex source of molecular information in oncology. Cellular DNA, urinary cfDNA, and ultrashort transrenal DNA are analytically distinct substrates, and extraction methods differ considerably in their ability to recover these fractions. Analytical studies demonstrate that preservation, urine volume, centrifugation, extraction chemistry, and fragment-size selectivity can substantially influence DNA recovery; however, these findings should not be interpreted as proof of clinical superiority of one extraction method over another.

Clinical evidence is currently strongest in urothelial carcinoma, where urinary DNA has been used for FGFR3 and TERT mutation detection, methylation analysis, genomic profiling, recurrence assessment, and treatment-response monitoring. Selected studies in renal, prostate, colorectal, endometrial, hepatocellular, and other malignancies support broader biological feasibility, although evidence outside urothelial cancer remains more heterogeneous and frequently derives from small or proof-of-concept cohorts.

Accordingly, standardization is an important but not the only barrier to clinical translation. Prospective multicenter validation, reproducibility across laboratories and extraction platforms, appropriate control of biological and clinical confounders, demonstration of added clinical utility, and cost-effectiveness remain essential. Urinary DNA analysis should therefore currently be regarded as a promising complementary liquid-biopsy strategy rather than a replacement for established diagnostic and surveillance pathways.

## Figures and Tables

**Table 1:** DNA extraction techniques summarized into several methodological categories.

Extraction Approach	Principle/Typical Target	Main Advantages	Main Limitations	Key Refs.
Conventional organic extraction	Phenol–chloroform extraction of mainly cellular/genomic DNA	Inexpensive, good recovery of relatively long DNA	Labor-intensive, toxic reagents, difficult to automate	[8]
Silica membrane spin-column extraction	DNA binds to silica under chaotropic conditions; widely used for cellular DNA and cfDNA	Standardized, simple, reproducible, good purity	Recovery can be poor for very short urinary cfDNA; multiple centrifugation steps	[4,9,16,18]
Magnetic bead/nanoparticle extraction	DNA or urinary cells bind to magnetic particles and are separated magnetically	Rapid, scalable, automatable, reduced hands-on time; suitable for genomic DNA and cfDNA	Recovery depends on bead chemistry and fragment size	[4,6,18,20]
Chelex/heat-based extraction	Heat lysis with chelating resin releases DNA from urinary sediment	Very simple, inexpensive, rapid	Lower purity; mainly appropriate for PCR rather than complex sequencing	[11]
Commercial urinary cfDNA kits	Optimized silica- or magnetic-based systems for low-concentration cfDNA	Convenient and standardized; compatible with PCR, ddPCR and NGS	Major differences in yield and fragment-size recovery among kits	[13,18,20]
Anion-exchange extraction (Q-Sepharose)	Negatively charged DNA binds to positively charged resin	High recovery of short urinary cfDNA compared with several conventional kits	Less standardized for routine clinical laboratories	[16]
Sequence-specific hybridization capture	Target DNA hybridizes to complementary probes attached to magnetic beads	Very high sensitivity; excellent recovery of extremely short, low-copy DNA; molecular enrichment during extraction	Target-specific, more technically complex, unsuitable when unbiased whole-genome recovery is required	[16,21]
High-molecular-weight DNA depletion/size-selective extraction	Large wild-type DNA is selectively removed to enrich short tumor-derived DNA	Can increase relative concentration of transrenal tumor DNA and improve mutation detection	May discard potentially useful longer tumor DNA; requires optimization	[30]
Automated multi-analyte extraction	Automated platforms simultaneously isolate DNA, RNA and sometimes proteins	High throughput, reproducibility, useful for biobanking and clinical workflows	Equipment and reagent costs; performance remains platform-dependent	[12]

**Table 2:** Representative analytical and clinical studies evaluating urinary DNA extraction and oncological applications.

Study	Evidence Type/Population	Urine Fraction and Volume	Preservation/Processing	Extraction Approach	Main Finding	Downstream Assay	Molecular Target/Biomarker	Principal Limitation
Bosschieter et al. [15]	Analytical/pre-analytical; 3 healthy volunteers in pilot phase; 10 bladder cancer and 10 NSCLC patients in clinical phase	Whole urine; 10 mL pilot aliquots; 4–10 mL patient aliquots	EDTA 40 mM, urine conditioning buffer, antibiotics or no preservative; RT/4/−20/−80 °C	Quick-DNA Urine Kit	Preservation and storage temperature substantially influenced recoverable methylated DNA.	qMSP	DNA methylation markers	Primarily a pre-analytical optimization study; limited ability to establish comparative clinical diagnostic performance.
Oreskovic et al. [16]	Analytical model using short DNA targets	Urinary cfDNA model; 25–150-nt targets	Controlled urine conditions including variable pH/background DNA	Wizard/GITC, Q-Sepharose, Norgen, QIAamp, MagMAX and hybridization capture	Hybridization capture and Q-Sepharose achieved the highest overall short-fragment recovery; conventional kits showed marked size bias.	Quantitative recovery/PCR analysis	Synthetic short DNA fragments, 25–150 nt	Synthetic/model DNA rather than a clinical cancer cohort; analytical recovery does not demonstrate diagnostic superiority.
Lee et al. [18]	Analytical; 10 healthy individuals	Urinary cfDNA; yield normalized per 1 mL urine	Multiple storage conditions; −70 °C + 10 mM EDTA performed best after prolonged storage	QIAamp Circulating NA, MagMAX, Norgen, Quick-DNA Urine	Recovery depended strongly on fragment size; Norgen and MagMAX performed favorably for 50–100-bp DNA.	Bioanalyzer fragment analysis	Total urinary cfDNA/fragment-size distribution	Small healthy-volunteer cohort with no cancer diagnostic endpoint; cost estimates were study-specific.
Augustus et al. [19]	Analytical/pre-analytical; 39 healthy volunteers and 14 patients with metastatic cancers across multiple subexperiments	Whole urine/cfDNA; experiment-specific volumes, including 12–75 mL	Fresh urine; Streck, UCM, CytoLyt or no preservative; different centrifugation and storage protocols	Quick-DNA Urine Kit	Collection timing, preservatives, temperature and centrifugation significantly affected cfDNA recovery and genomic DNA contamination.	ddPCR and fragment analysis	Total cfDNA and selected KRAS/PIK3CA targets	Individual experiments contained small numbers of subjects; findings require larger validation cohorts.
Eberhard et al. [27]	Analytical/translational; healthy controls and breast, colorectal and prostate cancer cohorts	Urinary cfDNA; variable volumes	Native, PAXgene or Streck stabilization; immediate and delayed processing	Automated QIAsymphony workflow	Native urinary cfDNA degraded rapidly, whereas stabilization preserved DNA suitable for several downstream molecular analyses.	dPCR, amplicon NGS, hybrid capture and shallow WGS	Cancer-associated mutations and genome-wide CNA	Heterogeneous cancer cohorts and downstream assays; some tumor signals were near or below analytical detection limits.
Hayashi et al. [40]	Clinical diagnostic study; 56 UTUC, 50 non-UC hematuria, 21 UC-surveillance and 26 control subjects	Urine supernatant cfDNA; 4–32 mL, median 12 mL	2000× *g* for 30 min; supernatant stored at −80 °C; additional 16,000× *g* clarification	QIAamp Circulating Nucleic Acid Kit	Mutation testing combined with cytology improved UTUC detection; study demonstrated clinical feasibility of urinary cfDNA.	ddPCR for TERT/FGFR3	TERT C228T/C250T and FGFR3 S249C	Relatively small UTUC cohort, age differences among groups, and limited follow-up; requires prospective large-scale validation.
Hentschel et al. [43]	Clinical diagnostic/technical comparison; 14 bladder cancer and 12 benign hematuria controls	Full void, pellet and supernatant; 30–40 mL collected; 15 mL fractionated	EDTA 40 mM; stored at −20 °C; 800× *g* for 10 min	Quick-DNA Urine for full void/supernatant; QIAamp DNA Mini for pellet	All fractions were informative; pellet produced the highest GHSR/MAL AUC (0.87), but cohort size limited strong diagnostic conclusions.	qMSP	GHSR/MAL and additional methylation markers	Very small cohort; authors considered the sample size appropriate for technical comparison but insufficient for strong diagnostic-accuracy conclusions.
Christensen et al. [48]	Clinical treatment-monitoring study; 92 MIBC patients	281 urine supernatants and 123 pellets; median 4 mL supernatant used for cfDNA extraction	Longitudinal collection before, during and after NAC	Validated urine cfDNA/cellular DNA workflow	Tumor DNA was detected before NAC in 89% of supernatants and 85% of pellets versus 43% of plasma; dynamics were associated with treatment response.	Patient-specific tumor-informed NGS	Patient-specific somatic tumor mutations	Tumor-informed personalized assay requiring prior tumor sequencing; findings require external prospective validation before routine implementation.
van den Helder et al. [56]	Clinical feasibility study; 42 endometrial cancer and 46 healthy controls	Full void, pellet and supernatant; 15 mL used for each extraction comparison	EDTA 40 mM; mailed and processed within 24–72 h; fractions stored at −20 °C	Quick-DNA Urine for full void/supernatant; QIAamp DNA Mini for pellet	Methylation was detectable in all fractions; full-void urine showed the strongest diagnostic discrimination for several markers.	qMSP	GHSR, SST, ZIC1 methylation	Feasibility study from a non-urological malignancy; results require larger prospective screening/diagnostic validation.
Smith et al. [55]	Clinical translational study; 91 patients with renal tumors; urine available in 37	Urine supernatant ± pellet; 30–50 mL	EDTA added within 1 h; samples stored at −80 °C	cfDNA extraction and personalized sequencing workflow	RCC showed low ctDNA shedding; tumor-derived urinary DNA was detectable in selected patients but requires more sensitive approaches.	Targeted/untargeted	Patient-specific somatic alterations/ctDNA	Limited urine availability and low tumor-DNA abundance; personalized testing was applied only to subsets of patients.
Wang et al. [50]	Clinical diagnostic/recurrence study; 436 UC/other urological disease patients and 79 healthy controls	Urinary cellular DNA	Study-specific urine processing	DNA extraction followed by methylation analysis	Two-gene methylation assay showed 85.2% sensitivity and 90.0% specificity and high performance for recurrence detection.	Real-time MSP	SOX1-OT and HIST1H4F methylation	Retrospective study; independent prospective multicenter validation and comparison with established diagnostic pathways are needed.

## Data Availability

No new data were created during this study.

## Abbreviations

The following abbreviations are used in this manuscript:

AUC	Area under the receiver operating characteristic curve
BC	Bladder cancer
cfDNA	Cell-free DNA
ctDNA	Circulating tumor DNA
ddPCR	Droplet digital polymerase chain reaction
DNA	Deoxyribonucleic acid
EDTA	Ethylenediaminetetraacetic acid
FGFR3	Fibroblast growth factor receptor 3
gDNA	Genomic DNA
MIBC	Muscle-invasive bladder cancer
MSP	Methylation-specific polymerase chain reaction
NAC	Neoadjuvant chemotherapy
NGS	Next-generation sequencing
NSCLC	Non-small-cell lung cancer
PCR	Polymerase chain reaction
qMSP	Quantitative methylation-specific polymerase chain reaction
RCC	Renal cell carcinoma
RNA	Ribonucleic acid
TERT	Telomerase reverse transcriptase
UC	Urothelial carcinoma
ucfDNA	Urinary cell-free DNA
UTUC	Upper tract urothelial carcinoma

## References

[1] van der Hel O.L., van der Luijt R.B., de Mesquita H.B.B., van Noord P.A., Slothouber B., Roest M., van der Schouw Y.T., Grobbee D.E., Pearson P.L., Peeters P.H. (2002). Quality and quantity of DNA isolated from frozen urine in population-based research. Anal. Biochem..

[2] Deelman L.E., van der Wouden E.A., Duin M., Henning R.H. (2002). A method for the ultra rapid isolation of PCR-ready DNA from urine and buccal swabs. Mol. Biol. Today.

[3] Kim C.S., Kim J.H., Kang D., Hong Y.C., Yoo K.Y. (2006). Gene amplification using DNA from human spot urine samples. Asian Pac. J. Cancer Prev..

[4] Siddiqui H., Nederbragt A.J., Jakobsen K.S. (2009). A solid-phase method for preparing human DNA from urine for diagnostic purposes. Clin. Biochem..

[5] Beermann A., Ghanjati F., Hermanns T., Poyet C., Pereira J., Fischer J., Wernet P., Santourlidis S. (2011). Methods for separate isolation of cell-free DNA and cellular DNA from urine—Application of methylation-specific PCR on both DNA fractions. Open Biomark. J..

[6] Shan Z., Zhou Z., Chen H., Zhang Z., Zhou Y., Wen A., Oakes K.D., Servos M.R. (2012). PCR-ready human DNA extraction from urine samples using magnetic nanoparticles. J. Chromatogr. B Anal. Technol. Biomed. Life Sci..

[7] Hilhorst M., Theunissen R., van Rie H., van Paassen P., Cohen Tervaert J.W. (2013). DNA extraction from long-term stored urine. BMC Nephrol..

[8] Ghatak S., Muthukumaran R.B., Nachimuthu S.K. (2013). A simple method of genomic DNA extraction from human samples for PCR-RFLP analysis. J. Biomol. Tech..

[9] El Bali L., Diman A., Bernard A., Roosens N.H.C., De Keersmaecker S.C.J. (2014). Comparative study of seven commercial kits for human DNA extraction from urine samples suitable for DNA biomarker-based public health studies. J. Biomol. Tech..

[10] Nauwelaerts S.J.D., Van Geel D., Delvoye M., De Cremer K., Bernard A., Roosens N.H.C., De Keersmaecker S.C.J. (2020). Selection of a noninvasive source of human DNA envisaging genotyping assays in epidemiological studies: Urine or saliva?. J. Biomol. Tech..

[11] Pankham A., Tatu T. (2020). Detecting HbE gene using DNA extracted from urine sediments by Chelex-plus-heating technique. J. Biomol. Tech..

[12] Barau C., Maillé P., Sirab N., Ghaleh B., Allory Y. (2022). Automated DNA, RNA, and protein extraction from urine for biobanking. J. Biomol. Tech..

[13] Streleckiene G., Reid H.M., Arnold N., Bauerschlag D., Forster M. (2018). Quantifying cell free DNA in urine: Comparison between commercial kits, impact of gender and inter-individual variation. BioTechniques.

[14] Lecamwasam A., Sexton-Oates A., Carmody J., Ekinci E.I., Dwyer K.M., Saffery R. (2018). DNA methylation profiling of genomic DNA isolated from urine in diabetic chronic kidney disease: A pilot study. PLoS ONE.

[15] Bosschieter J., Bach S., Bijnsdorp I.V., Segerink L.I., Rurup W.F., Van Splunter A.P., Bahce I., Novianti P.W., Kazemier G., van Moorselaar R.J.A. (2018). A protocol for urine collection and storage prior to DNA methylation analysis. PLoS ONE.

[16] Oreskovic A., Brault N.D., Panpradist N., Lai J.J., Lutz B.R. (2019). Analytical comparison of methods for extraction of short cell-free DNA from urine. J. Mol. Diagn..

[17] Li P., Ning J., Luo X., Du H., Zhang Q., Zhou G., Du Q., Ou Z., Wang L., Wang Y. (2019). New method to preserve the original proportion and integrity of urinary cell-free DNA. J. Clin. Lab. Anal..

[18] Lee E.Y., Lee E.J., Yoon H., Lee D.H., Kim K.H. (2020). Comparison of four commercial kits for isolation of urinary cell-free DNA and sample storage conditions. Diagnostics.

[19] Augustus E., Van Casteren K., Sorber L., van Dam P., Roeyen G., Peeters M., Vorsters A., Wouters A., Raskin J., Rolfo C. (2020). The art of obtaining a high yield of cell-free DNA from urine. PLoS ONE.

[20] Lin S.Y., Luo Y., Marshall M.M., Johnson B.J., Park S.R., Wang Z., Su Y.-H. (2021). A new method for improving extraction efficiency and purity of urine and plasma cell-free DNA. Diagnostics.

[21] Oreskovic A., Lutz B.R. (2021). Ultrasensitive hybridization capture: Reliable detection of <1 copy/mL short cell-free DNA from large-volume urine samples. PLoS ONE.

[22] Jordaens S., Arora A., MacDonald K.W., Wood C., Hendrickx J.O., Zwaenepoel K., Deben C., Tjalma W., Pauwels P., Beyers K. (2023). UAS™—A urine preservative for oncology applications. Cancers.

[23] Nel I., Münch C., Shamkeeva S., Heinemann M.L., Isermann B., Aktas B. (2023). The challenge to stabilize, extract and analyze urinary cell-free DNA (ucfDNA) during clinical routine. Diagnostics.

[24] Ruppert T., Roth A., Kollmeier J., Mairinger T., Frost N. (2023). Cell-free DNA extraction from urine of lung cancer patients and healthy individuals: Evaluation of a simple method using sample volume up-scaling. J. Clin. Lab. Anal..

[25] Bhambhani C., Kang Q., Hovelson D.H., Sandford E., Olesnavich M., Dermody S.M., Wolfgang J., Tuck K.L., Brummel C., Bhangale A.D. (2024). CtDNA transiting into urine is ultrashort and facilitates noninvasive liquid biopsy of HPV+ oropharyngeal cancer. JCI Insight.

[26] Sandberg F., Kueng N., Largiadèr C.R., Amstutz U. (2025). Evaluation of variability in cell-free DNA extraction efficiency from plasma and urine and spike-in normalization. Sci. Rep..

[27] Eberhard A., Moser T., Ziegler L., Vlachos G., Loibner M., Bauernhofer T., Balic M., Gerger A., Dandachi N., Beichler C. (2025). Evaluation of urinary cfDNA workflows for the molecular profiling of malignant disease. iScience.

[28] Mauger F., Oussada N., Horgues C., Eberhard A., Oman L., Gou M., Lumière K., Mancarella-Langer D., Voss T., McGinn S. (2026). A comprehensive urine workflow enables robust methylation and fragmentation analysis of cell-free DNA. Sci. Rep..

[29] Su Y.-H., Wang M., Brenner D.E., Ng A., Melkonyan H., Umansky S., Syngal S., Block T.M. (2004). Human urine contains small, 150 to 250 nucleotide-sized, soluble DNA derived from the circulation and may be useful in the detection of colorectal cancer. J. Mol. Diagn..

[30] Su Y., Song J., Wang Z., Wang X., Wang M., Brenner D.E., Block T.M. (2008). Removal of high-molecular-weight DNA by carboxylated magnetic beads enhances the detection of mutated K-ras DNA in urine. Ann. N. Y. Acad. Sci..

[31] Su Y.H., Wang M., Brenner D.E., Norton P.A., Block T.M. (2008). Detection of mutated K-ras DNA in urine, plasma, and serum of patients with colorectal carcinoma or adenomatous polyps. Ann. N. Y. Acad. Sci..

[32] Rieger-Christ K.M., Medina A., Lee P.J., Rieger-Christ K.M., Mourtzinos A., Lee P.J., Libertino J.A., Summerhayes I.C. (2003). Identification of fibroblast growth factor receptor 3 mutations in urine sediment DNA samples complements cytology in bladder tumor detection. Cancer.

[33] Szarvas T., Kovalszky I., Bedi K., Szendroi A., Majoros A., Riesz P., Füle T., László V., Kiss A., Romics I. (2007). Deletion analysis of tumor and urinary DNA to detect bladder cancer: Urine supernatant versus urine sediment. Oncol. Rep..

[34] Wang K., Liu T., Liu C., Meng Y., Yuan X., Liu L., Ge N., Liu J., Wang C., Ren H. (2015). TERT promoter mutations and TERT mRNA but not FGFR3 mutations are urinary biomarkers in Han Chinese patients with urothelial bladder cancer. Oncologist.

[35] Ward D.G., Baxter L., Gordon N.S., Ott S., Savage R.S., Beggs A.D., James J.D., Lickiss J., Green S., Wallis Y. (2016). Multiplex PCR and next generation sequencing for the non-invasive detection of bladder cancer. PLoS ONE.

[36] Togneri F.S., Ward D.G., Foster J.M., Devall A.J., Wojtowicz P., Alyas S., Vasques F.R., Oumie A., James N.D., Cheng K.K. (2016). Genomic complexity of urothelial bladder cancer revealed in urinary cfDNA. Eur. J. Hum. Genet..

[37] Lee D.H., Yoon H., Park S., Kim J.S., Ahn Y.-H., Kwon K., Lee D., Kim K.H. (2018). Urinary exosomal and cell-free DNA detects somatic mutation and copy number alteration in urothelial carcinoma of bladder. Sci. Rep..

[38] Russo I.J., Ju Y., Gordon N.S., Zeegers M.P., Cheng K., James N.D., Bryan R.T., Ward D.G. (2018). Toward personalised liquid biopsies for urothelial carcinoma: Characterisation of ddPCR and urinary cfDNA for the detection of the TERT 228. Bladder Cancer.

[39] Stasik S., Salomo K., Heberling U., Froehner M., Sommer U., Baretton G.B., Ehninger G., Wirth M.P., Thiede C., Fuessel S. (2019). Evaluation of TERT promoter mutations in urinary cell-free DNA and sediment DNA for detection of bladder cancer. Clin. Biochem..

[40] Hayashi Y., Fujita K., Matsuzaki K., Matsushita M., Kawamura N., Koh Y., Nakano K., Wang C., Ishizuya Y., Yamamoto Y. (2019). Diagnostic potential of TERT promoter and FGFR3 mutations in urinary cell-free DNA in upper tract urothelial carcinoma. Cancer Sci..

[41] Ward D.G., Gordon N.S., Boucher R.H., Pirrie S.J., Baxter L., Ott S., Silcock L., Whalley C.M., Stockton J.D., Beggs A.D. (2019). Targeted deep sequencing of urothelial bladder cancers and associated urinary DNA: A 23-gene panel with utility for non-invasive diagnosis and risk stratification. BJU Int..

[42] Ou Z., Li K., Yang T., Dai Y., Chandra M., Ning J., Wang Y., Xu R., Gao T., Xie Y. (2020). Detection of bladder cancer using urinary cell-free DNA and cellular DNA. Clin. Transl. Med..

[43] Hentschel A.E., Nieuwenhuijzen J.A., Bosschieter J., van Splunter A.P., Lissenberg-Witte B.I., van der Voorn J.P., Segerink L.I., van Moorselaar R.J.A., Steenbergen R.D. (2020). Comparative analysis of urine fractions for optimal bladder cancer detection using DNA methylation markers. Cancers.

[44] Hayashi Y., Fujita K., Matsuzaki K., Eich M.-L., Tomiyama E., Matsushita M., Koh Y., Nakano K., Wang C., Ishizuya Y. (2020). Clinical significance of hotspot mutation analysis of urinary cell-free DNA in urothelial bladder cancer. Front. Oncol..

[45] Hentschel A.E., Helder R.v.D., van Trommel N.E., van Splunter A.P., van Boerdonk R.A.A., van Gent M.D.J.M., Nieuwenhuijzen J.A., Steenbergen R.D.M. (2021). The origin of tumor DNA in urine of urogenital cancer patients: Local shedding and transrenal excretion. Cancers.

[46] Jain M., Kamalov D., Tivtikyan A., Balatsky A., Samokhodskaya L., Okhobotov D., Kozlova P., Pisarev E., Zvereva M., Kamalov A. (2021). Urine TERT promoter mutations-based tumor DNA detection in patients with bladder cancer: A pilot study. Mol. Clin. Oncol..

[47] Li N., Wang L., Liang H., Lin C., Yi J., Yang Q., Luo H., Luo T., Zhang L., Li X. (2022). Detecting and monitoring bladder cancer with exfoliated cells in urine. Front. Oncol..

[48] Christensen E., Nordentoft I., Birkenkamp-Demtröder K., Elbæk S.K., Lindskrog S.V., Taber A., Andreasen T.G., Strandgaard T., Knudsen M., Lamy P. (2023). Cell-free urine and plasma DNA mutational analysis predicts neoadjuvant chemotherapy response and outcome in patients with muscle-invasive bladder cancer. Clin. Cancer Res..

[49] Goel A., Tura B.J., Stockton J.D., Tovey N., Ames L., Beggs A.D., Zeegers M.P., James N.D., Cheng K.K., Bryan R.T. (2025). Detection of genome-wide methylation changes in bladder cancer by long-read sequencing of urinary DNA. Clin. Epigenet..

[50] Wang D., Ai L., Tan H., Yan Y., Ye H., He M., Huang C.-H., Chen P., Liang Y., Wang R. (2025). A urinary DNA methylation assay using two genes enables noninvasive detection and prognostic prediction in urothelial carcinoma. Sci. Rep..

[51] Crisafulli G., Mussolin B., Cassingena A., Montone M., Bartolini A., Barault L., Martinetti A., Morano F., Pietrantonio F., Sartore-Bianchi A. (2019). Whole exome sequencing analysis of urine trans-renal tumour DNA in metastatic colorectal cancer patients. ESMO Open.

[52] Casadio V., Calistri D., Salvi S., Gunelli R., Carretta E., Amadori D., Silvestrini R., Zoli W. (2013). Urine cell-free DNA integrity as a marker for early prostate cancer diagnosis: A pilot study. BioMed Res. Int..

[53] Xia Y., Huang C.-C., Dittmar R., Du M., Wang Y., Liu H., Shenoy N., Wang L., Kohli M. (2016). Copy number variations in urine cell free DNA as biomarkers in advanced prostate cancer. Oncotarget.

[54] Fujii T., Barzi A., Sartore-Bianchi A., Cassingena A., Siravegna G., Karp D.D., Piha-Paul S.A., Subbiah V., Tsimberidou A.M., Huang H.J. (2017). Mutation-enrichment next-generation sequencing for quantitative detection of KRAS mutations in urine cell-free DNA from patients with advanced cancers. Clin. Cancer Res..

[55] Smith C.G., Moser T., Mouliere F., Field-Rayner J., Eldridge M., Riediger A.L., Chandrananda D., Heider K., Wan J.C.M., Warren A.Y. (2020). Comprehensive characterization of cell-free tumor DNA in plasma and urine of patients with renal tumors. Genome Med..

[56] van den Helder R., Wever B.M.M., van Trommel N.E., van Splunter A.P., Mom C.H., Kasius J.C., Bleeker M.C.G., Steenbergen R.D.M. (2020). Non-invasive detection of endometrial cancer by DNA methylation analysis in urine. Clin. Epigenet..

[57] Bach S., Paulis I., Sluiter N.R., Tibbesma M., Martin I., van de Wiel M.A., Tuynman J.B., Bahce I., Kazemier G., Steenbergen R.D.M. (2021). Detection of colorectal cancer in urine using DNA methylation analysis. Sci. Rep..

[58] Kim A.K., Hamilton J.P., Lin S.Y., Chang T.-T., Hann H.-W., Hu C.-T., Lou Y., Lin Y.-J., Gade T.P., Park G. (2022). Urine DNA biomarkers for hepatocellular carcinoma screening. Br. J. Cancer.

[59] Ward D.G., Bryan R.T., Chaudhuri A.A., Hadfield J., Perez-Boza J., Steenbergen R.D.M., Vega D.M., Whiting J., Wyatt A.W., Dyrskjøt L. (2026). Unlocking the potential of urine-based liquid biopsy through improved reporting and standardization. Nat. Rev. Cancer.

[60] (2026). Molecular In Vitro Diagnostic Examinations—Requirements and Recommendations for Pre-examination Processes for Urine and Other Body Fluids—Isolated Cell-Free DNA. 1st ed..

